# For girls and women (4GW) HPV RCT protocol: a crowdsourced, pragmatic stepped-wedge cluster randomized trial to improve uptake of HPV vaccination and screening among mother-daughter dyads in Nigeria

**DOI:** 10.1186/s13012-025-01428-5

**Published:** 2025-05-01

**Authors:** Juliet Iwelunmor, Agatha E. Wapmuk, Ekenechukwu Kokelu, Temitope Ojo, Olufunto Olusanya, Titilola Gbaja-Biamila, Folahanmi T. Akinsolu, Adesola Z. Musa, Hong Xian, Olunike R. Abodunrin, Peter Kalulu, Angel Obiorah, Maria Afadapa, Nkiruka Obodoechina, Ucheoma Nwaozuru, Onyeka Anikamadu, Jennifer Smith, Benedict N. Azuogu, Kayode Ajenifuja, Mengmeng Jia, Assanatou Bamogo, Abdulhammed Babatunde, Jason J. Ong, Lei Zhang, Zhuoru Zou, Collins O. Airhihenbuwa, Joseph D. Tucker, Oliver C. Ezechi

**Affiliations:** 1 Division of Infectious Diseases, Washington University School of Medicine in St. Louis, 4523 Clayton Ave., MSC 8051 - 0043 - 15, St. Louis , MO 63110 USA; 2 Clinical Sciences Department, Nigerian Institute of Medical Research, Lagos, Nigeria; 3 Center for Reproduction and Population Health Studies, Nigerian Institute of Medical Research, Lagos, Nigeria; 4 Implementation Science Department, Feinburg School of Medicine, Northwestern University, Chicago, USA; 5 College for Public Health & Social Justice, Saint Louis University, Saint Louis, MO USA; 6 Department of Biostatistics and Epidemiology, Nanjing Medical University, Jiangsu, China; 7 Department of Public Health, Lead City University, Oyo, Nigeria; 8 Department of Implementation Science, Division of Public Health Sciences, Wake Forest School of Medicine, Winston-Salem, NC USA; 9 Central Clinical School, Monash University, Melbourne, Australia; 10 Institute of Global Health and Infectious Diseases, University of North Carolina at Chapel Hill, Chapel Hill, NC USA; 11 Department of Anaesthesia, Ebonyi State University, Abakaliki, Ebonyi State Nigeria; 12 Department of Obstetrics and Gynaecology, Obafemi Awolowo University, Osun State, Ile-Ife, Nigeria; 13 Department of Medicine, University of North Carolina at Chapel Hill, Chapel Hill, NC USA; 14 Department of Medicine and Surgery, Faculty of Clinical Sciences, College of Medicine, University of Ibadan, Oyo, Nigeria; 15 Health Policy and Behavioral Sciences, School of Public Health, Georgia State University, Atlanta, GA USA; 16 School of Public Health, Xi’an Jiaotong University XJTU · Health Science Centre, Xi’an, Shaanxi China; 17 Melbourne Sexual Health Centre, Alfred Health, Melbourne, Australia; 18 Faculty of Infectious and Tropical Diseases, London School of Hygiene and Tropical Medicine, London, UK; 19 National Institute of Pathogen Biology, Chinese Academy of Medical Sciences, Beijing, 102629 China

## Abstract

**Background:**

Expanding human papillomavirus (HPV) vaccination for girls and HPV self-collection for women can reduce the global burden of cervical cancer. However, HPV vaccination and self-collection services are rarely implemented simultaneously in mother-daughter dyads, leaving a critical gap in cervical cancer prevention. From 2023 to 2024, a community-engaged model for combined HPV vaccination and screening was co-designed using crowdsourcing open calls and designathons with mother-daughter teams and pilot-tested by trained research facilitators. This study explores the impact of this crowdsourced, community-engaged mother-daughter campaign and implementation strategy bundle on HPV vaccination among girls and HPV screening among their mothers in Nigeria over 6 months in 18 Nigerian local government areas (LGAs).

**Methods:**

A hybrid effectiveness-implementation type II pragmatic stepped-wedge cluster randomized control trial has been employed to the effectiveness of an implementation strategy bundle; a crowdsourced, tailored, community-engaged, mother-daughter HPV campaign on increasing uptake of HPV vaccination among girls aged 9–14 and HPV screening uptake among women aged 30–49 in Nigeria. The mother-daughter campaign will be tailored to local sites and conducted among 612 mother-daughter dyads (1,224 participants) recruited from 18 LGAs in six geopolitical zones of Nigeria. Trained community health workers will collect baseline data and implement a mother-daughter campaign that will provide education on cervical cancer control and access to onsite services for HPV vaccination and screening in a private area while engaging mothers and daughters simultaneously to increase uptake of the services. A mixed-methods evaluative and iterative assessment will be conducted using Proctor’s Implementation Outcomes Framework and the PEN- 3 cultural model. The primary outcomes are the uptake of HPV preventive measures—HPV vaccination (one dose) among girls (ascertained by onsite clinical records of vaccine uptake) and HPV self-collection completion among mothers (ascertained by laboratory receipt of self-collected specimens) within six months of trial enrollment. Pre-post effectiveness and cost of study components are embedded in the implementation and sustainment phases, compared to pre-implementation data assessed for each LGA.

**Discussion:**

This study is a unique dyadic intervention focused on both girls and their mothers or female caregivers to drive cervical cancer control in Africa. Findings have the potential to inform local and global policies aimed at reducing the cervical cancer burden in African countries like Nigeria, eliminating missed opportunities by closing the research-to-translation gap. The protocol was registered with clinicaltrials.gov under registration NCT06728085.

**Supplementary Information:**

The online version contains supplementary material available at 10.1186/s13012-025-01428-5.

Contributions to the literature The study will be the first study to test whether a combined mother-daughter HPV campaign, tailored to local contexts, with education on cervical cancer and onsite services provided (i.e. access to HPV vaccination and screening in private spaces), increases HPV vaccination among daughters and HPV self-collection among mothers recruited in Nigeria.

This is the first application of crowdsourcing and participatory approaches to optimize cervical cancer control among mother-daughter dyads in Nigeria.

Our study is designed to provide decision-makers with evidence, including the cost-effectiveness of a final combined implementation strategy bundle for HPV vaccination and screening among mother-daughter dyads in Nigeria.

## Introduction

Eliminating invasive cervical cancer in African countries is a challenge. In 2022, there were 13,676 new cervical cancer cases and 7,093 associated deaths in Nigeria, making cervical cancer the second most common cancer in women [1]. Cervical cancer is caused by persistent infection with one of 13 carcinogenic human papillomavirus (HPV) types [2]. The World Health Organization has called for the global elimination of cervical cancer to achieve 90–70–90 targets by 2030, whereby 90% of girls are fully vaccinated for HPV by age 15, 70% of women are screened with high-performance tests by the age of 35 years and again by age 45, and 90% of women with cervical pre-cancer or cancer are treated [2, 3]. Multiple evidence-based strategies for promoting HPV-based prevention methods have been developed, including strategies targeting health, school, family, community, and policy contexts [4–6]. Yet barriers to the uptake of these methods exist at multiple levels, including individual (low-risk perception levels), dyadic/social (poor support), and community/structural (limited [6]access) systems [7–10]. HPV vaccination of girls and HPV self-collection for cervical cancer screening among mothers (or caregivers, guardians), particularly mother/daughter dyads within family and community systems, may be able to overcome some of these barriers [11–16]. However, both vaccination and HPV self-collection services are still largely inaccessible and have limited input from end-users, especially local girls and their mothers. Nigeria is unlikely to eliminate cervical cancer unless HPV prevention methods are tailored and implemented in culturally compelling, cost-effective and sustainable ways [3]. One approach to expanding the implementation of HPV prevention strategies is crowdsourcing.

Crowdsourcing has a group collectively solve a problem and then implement proposed solutions [17]. This encompasses various participatory activities, including open calls to obtain ideas, designathons, and participatory learning communities [18, 19]. Learning community groups are structured, collective, problem-solving, participatory strategies that convene participants around a common problem, working together to learn best practices and exchange experiences informed by quality improvement methods [20]. Crowdsourced implementation strategies can expand the acceptability, feasibility, and sustainability of HPV-prevention methods by leveraging end-user expertise directly to design and implement evidence-based strategies relevant to the local context [21, 22]. Researchers, community members, and other relevant actors work together to learn from each other, generate knowledge, and support change in a participatory manner [23]. These participatory strategies also advance health equity by centering relationships between researchers and those with lived experience to improve the pathway between research and practice necessary for eliminating persistent inequities in health [24]. Crowdsourcing approaches have been used before in Nigeria to increase HIV and STI test uptake among Nigerian youth [21].

Nigeria has programs and infrastructure in place to support the combined implementation of HPV vaccination and HPV self-collection among mother-daughter dyads [3]. Recently, to immunize 16 million girls by 2025 with a single dose of HPV vaccine, Nigeria launched a nationwide rollout of HPV vaccination in October 2023 when 15 states plus the Federal Capital made HPV vaccination available to approximately 5.3 million girls in an initial phase [25]. Phase 2 launched in May 2024 and over seven million girls in 21 states were vaccinated [25]. HPV vaccination is classified as a best buy for non-communicable diseases and is considered a highly cost-effective and affordable intervention for cervical cancer prevention [26]. Likewise, the 2019 Nigerian Society of Obstetrics and Gynecology guidelines [27], as well as the Nigerian National Strategic Plan for cancer, recommend routine HPV screening for women by ages 30–65 years old, including the early detection of asymptomatic pre-cancerous lesions and prompt treatment to prevent cervical cancers [3, 28]. Nonetheless, attempts to implement HPV vaccination and HPV screening in real-world settings in Nigeria have focused on standalone interventions implemented separately with girls or women as with the national rollout of HPV vaccination. Few studies have investigated the strategies necessary to integrate both HPV vaccination and HPV screening with mother-daughter dyads simultaneously, despite the availability of evidence suggesting its feasibility [16, 29]. Combined HPV vaccination and screening have the potential to improve the adoption of cervical cancer prevention interventions [16]. Such an approach capitalizes on reciprocal learning between mothers and daughters and facilitates the mutual reinforcement of health prevention practices and behaviors [12, 30–32]. Notably, dyadic approaches align with many cultures where parents make important health decisions for young girls [33–35]. Given the high incidence and mortality rates of cervical cancer in Nigeria, efforts to equitably increase the uptake of HPV vaccination and HPV screening using culturally compelling strategies are needed.

Known locally as the For Girls and Women (4GW) study, the study builds on our team’s participatory implementation science research for HIV prevention in Nigeria [36]. We organized a participatory HIV research study called 4 Youth by Youth study [22]. In the pre-implementation phase (Years 1–2), we conducted a crowdsourcing open call and used designathons and innovation bootcamps (similar to our HIV research) to develop implementation strategies for increasing HPV vaccination and HPV screening among mother-daughter dyads [37]. We iteratively piloted and refined the strategies using participatory learning communities and created and finalized study materials similar to our prior research [21]. Preliminary findings demonstrate that an implementation strategy [38] bundle that combines a mother-daughter campaign, tailored and adapted to local contexts, with education on cervical cancer and access to onsite services (i.e. access to HPV vaccination and HPV screening in private spaces) that engages mothers and daughters as an implementation strategy bundle, increases the uptake of HPV vaccination among Nigerian girls and HPV screening among their mothers. Here, we describe the study protocol for the stepped-wedge cluster randomized control hybrid type II trial to be implemented by trained community health workers (CHWs) in 18 local government areas (LGAs) with 612 mother/daughter dyads. The purpose of the trial is to evaluate the effectiveness and cost-effectiveness of the final combined implementation strategy bundle for HPV vaccination and screening among mother/daughter dyads in Nigeria.

## Methods

This study is a collaboration between the Washington University School of Medicine, the University of North Carolina at Chapel Hill, Monash University, Wake Forest University School of Medicine, and the Nigerian Institute of Medical Research (NIMR). Established in 1977 as Nigeria's premier national medical research institute under the Ministry of Health, NIMR is a hub for research, capacity building, and collaborative efforts to advance national development. Its mission encompasses basic, applied, and implementation science research on infectious and non-communicable diseases of public health relevance in Nigeria. This work is funded by the National Cancer Institute, with pre-implementation and implementation phases focused on collaborative, community-engaged strategies for cervical cancer and HPV prevention.

This hybrid effectiveness type II [39] stepped-wedge cluster randomized trial will test the hypothesis that a combined mother-daughter campaign, tailored and adapted to local contexts, with education on cervical cancer and onsite services provided (i.e. access to HPV vaccination and screening in private spaces), increases HPV vaccination among girls and HPV self-collection among mothers. This study was conducted and reported in accordance with the CONSORT extension for Stepped-Wedge Cluster Randomized Trials (SW-CRTs) to ensure transparency, completeness, and methodological rigor [40]. The CONSORT extension provides specific guidelines for reporting SW-CRTs, including the justification for the use of the stepped-wedge design, details of the cluster randomization process, sequence generation, and allocation concealment. (See Supplementary File 1 for the CONSORT checklist).

Mothers are female caregivers such as biological mothers, aunts, grandmothers, or guardians. We selected a pragmatic type II hybrid effectiveness design because it allows for a simultaneous assessment of the intervention implementation effectiveness and clinical outcomes assessment [39]. The study will be conducted among 612 mother-daughter dyads (*n* = 612 × 2 = 1,224 participants) recruited from 18 LGAs to a stepped-wedge randomized control trial of the crowdsourced intervention versus usual care. All mother-daughter dyads will be followed for 12 months. The Proctor’s Implementation Outcomes Framework [41] and the PEN- 3 cultural model [42] will guide the analysis and evaluation of the implementation process. We will recruit and train one CHW per LGA (*n* = 18) with one supervisor per LGA (n = 18) to assist with overall study implementation. Before participant recruitment, all CHWs will receive training on the final intervention, good clinical practice and human subject research. CHWs are key partners [43] that will recruit mother-daughter dyads meeting the eligibility criteria, identify barriers to engagement and participation, tailor and adapt the mother-daughter campaign to include access to onsite services for HPV self-collection kits, screen mothers and vaccination of girls by study nurses. CHWs will transport self-collected cervical samples to the NIMR laboratory, provide test results, link mothers to treatment for positive test results and retain participants overtime to advance efforts to eliminate cervical cancer in their LGAs. The trial is designed as a pragmatic study to closely mirror real-world conditions for on-going HPV prevention services in Nigeria.

### Conceptual framework

The study design, methods, and analysis integrate implementation science frameworks, specifically Proctor’s implementation framework [41], with a community-engaged approach informed by the PEN- 3 cultural model [42] and social learning principles [44, 45]. The PEN- 3 focuses on Perceptions (beliefs), Enablers (resources), and Nurturers (peers, family, community support) impacting health within a cultural context considering the Positive, Existential and Negative factors [46]. It has been used to explore health needs and research questions, determine study design, recruit participants, and implement and disseminate health research findings congruent with participant beliefs and perceptions [46]. Researchers have used the PEN- 3 model to design cervical cancer interventions among minority populations in the US [47]. We will also use principles from social learning to guide our participatory learning communities [45]. Social learning theory suggests that new behaviors can be supported by observing and emulating others [45]. Social learning principles have enhanced the implementation and sustainability of evidence-based interventions in real-world settings, including mother-daughter interventions [48, 49]. Finally, Proctor’s implementation outcomes framework guides the evaluation of implementation success [41]. The framework will guide our evaluation of distinct implementation outcomes (acceptability, adoption, appropriateness, cost, feasibility, fidelity, penetration, and sustainability) to determine whether success or failure was due to the intervention or the implementation process [41].

### Study setting aims and approach

#### Setting

**Fig. 1:**
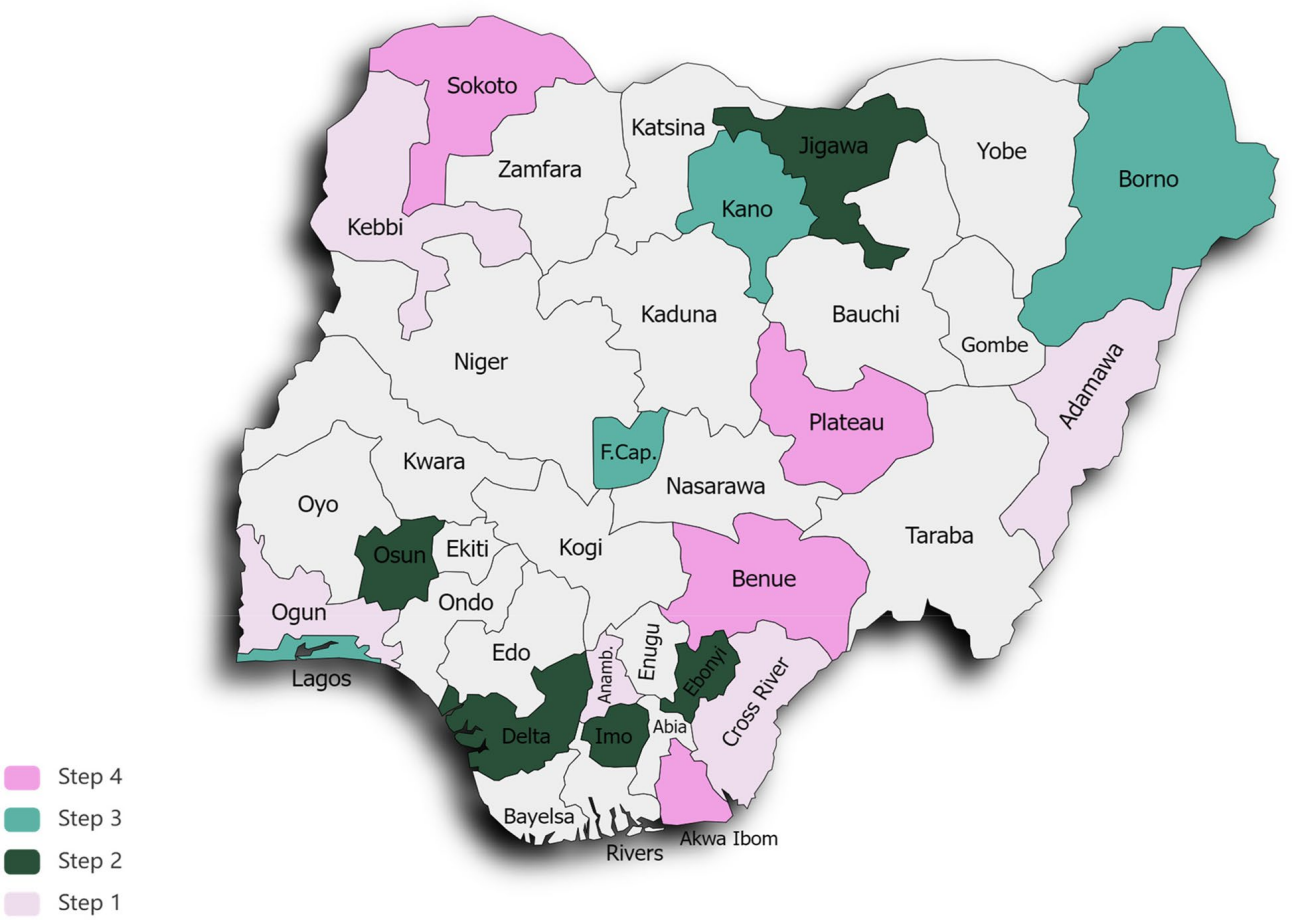
Map of Nigeria and 4GW study sites

Nigeria’s population is estimated at over 200 million, of which 49.3% are female [50, 51]. The country has 36 states mapped into six geopolitical zones with a Federal Capital territory [50]. There are also 374 distinct ethnic groups and indigenous languages spoken throughout its 36 states and Federal Capital Territory [50]. Data from the most recent Demographic Health Survey (2018) estimates that the median age at first sexual intercourse among women is 17 years of age and first marriage at 19 years [52]. Nearly half of women aged 25–49 give birth for the first time before age 21, while 19% of teenage women aged 15–19 have begun childbearing [52]. The Literacy rate in Nigeria is 60% [53]. Also, 65% of women aged 15–49 are employed or self-employed [52], while the monthly minimum wage is 30,000 naira ($18.4) [54]. Although HPV vaccine coverage has increased with the nationwide rollout at 75% [55], it remains notably below the 90% target needed to eliminate cervical cancer as a public health concern. Additionally, despite recommendations for routine cervical cancer screening for women aged 30–49, cervical cancer screening coverage remains under 10% [56, 57]. HPV self-sampling, a potentially accessible method for increasing screening uptake, is also not yet widely implemented or routinely practiced in Nigeria. Organized cervical cancer screening programs are limited, particularly in community settings where most individuals receive healthcare. Our partnership with the Nigerian Institute for Medical Research is critical for enhancing the feasibility of the study. As the key research hub within the Nigerian Ministry of Health, NIMR is well-positioned to design and deliver the intervention across the 18 recruited local government areas within the six geopolitical zones of Nigeria (See Fig. 1).

### Participants

Our stepped-wedge cluster-randomized trial will consist of two types of participants: 1) mother-daughter dyads recruited from 18 randomly selected LGAs across six geo-political zones in Nigeria with access to NIMR-affiliated clinical staff and 2) CHWs (one per LGA) and supervisors (one per LGA) implementing the intervention. Study eligibility criteria are described in Table 1.

**Table 1:** Inclusion and exclusion criteria

Inclusion criteria (mother-daughter dyads)	• Both the mothers'/caregivers’ (between ages 30–49 years) and daughters'(Between 9 to 14 years) willingness to participate in the study• All participants must agree to informed consent in English or Pidgin English, Nigeria's second most common language.• Parental or guardian’s cell phone number for follow-up
Exclusion criteria (mother-daughter dyads)	• Inability to comply with the study protocol • Girls with recent vaccination for HPV or mothers recently screened for cervical cancer (within the last 5 years) • Illness, cognitive impairment, or threatening behaviour with acute risk to self or others

### Evidence-based intervention

We operationalize our evidence-based intervention as HPV vaccination for girls aged 9–14 and HPV screening for women aged 30–49, following national guidelines. Although these guidelines support the promotion of HPV vaccination and screening as evidence-based practices, they remain poorly implemented in routine community settings in Nigeria [27, 28]. Despite nationwide rollout, HPV vaccination uptake among adolescents falls short of the global target of 90% coverage by 2030. Similarly, organized national screening programs are lacking, limiting screening access among women [3]. While mother-daughter interventions promoting vaccination for young girls and screening for their mothers are feasible and effective in other contexts, they are underutilized in Nigeria [16]. By focusing on vaccination and screening, this study aims to enhance dyadic uptake of HPV prevention strategies to reduce cervical cancer. Additionally, we will examine barriers to dyadic participation, recognizing that service delivery points for vaccination and screening are often separate, which may impact uptake. Addressing these barriers is crucial for developing effective, community-centered HPV prevention interventions for girls and women.

#### Crowdsourced implementation strategy bundle

We will deploy a 5-component bundle, crowdsourced and designed by mother-daughter teams and pilot-tested by trained research facilitators (see Additional file 1). Our combined HPV campaign was developed through an iterative, multi-step process [36, 58], including a crowdsourcing open call, a 48-h designathon, an innovation bootcamp, and pilot-testing of seven team ideas in Nigeria. Four teams participated in Phase 1 of the nationwide rollout of HPV vaccination, where their interventions were implemented to coincide with free access to single-dose HPV vaccinations, while three teams participated in Phase 2 of the nationwide rollout of free HPV vaccination for girls. Our final crowdsourced implementation strategy bundle combines elements from the top pilot campaign in Phase 1 and Phase 2 to create the 4GW mother-daughter campaign that includes:

- Tailor and adapt plans to context: Mother-Daughter Day campaigns will be tailored and adapted to the local contexts within the 18 LGA with our simplified PLAN [59] building block tool used as a guide to document and track adaptations made across the 18 sites and reported using FRAME-IS after intervention.
- Develop partnerships: We will identify and train supervisors at NIMR-affiliated clinical sites for leadership roles and identify and prepare CHWs (1 per LGA) to build a 4GW Mother-Daughter Day coalition for cervical cancer control. Supervisors selected will be trained physicians who can provide cervical cancer treatment for mothers who test positive. Supervisors will also identify CHWs with experience conducting community health research and intentions to remain in the community for at least 12 months. A 4GW ambassador group of community research facilitators from the pilot test will support local adaptation and implementation.
- Capacity strengthening: We will develop and distribute educational materials for the mother-daughter Day HPV campaign, conduct ongoing training and outreach visits, and create a monthly participatory learning collaborative to reflect and document implementation phenomena periodically.
- Engage mothers and daughters with onsite access to services: We have previously involved mothers and daughters as teams in developing and pilot-testing the final HPV campaign. We will continue to prepare them to be active participants throughout the implementation process of the onsite services for HPV (i.e. access to study nurses for HPV vaccinations and HPV self-collection in a private area), conducting pre-post surveys that explore their perceptions of the campaigns, hurdles and opportunities for using the campaigns to increase uptake of cervical cancer prevention services among mother-daughter dyads in Nigeria.
- Use of evaluative and iterative strategies: Before implementation, we will assess readiness among supervisors and research facilitators to identify barriers and facilitators with implementation. We will develop and implement tools for quality monitoring and use findings to finalize an implementation blueprint for mother-daughter HPV campaigns for cervical cancer control in Nigeria.

Table 2 describes the implementation bundle using Proctor’s and colleagues’ reporting recommendations [60].

**Table 2:** Implementation strategy specification

Domain	Strategy: 4GW Mother-Daughter Day HPV campaign
Actor	- Mother-daughter dyads, CHWs, and supervisors
Action	- Tailor and adapt the Mother-Daughter Day HPV campaign - Develop partner interrelationships for implementation - Train and educate partners - Engage mothers and daughters with onsite access to HPV services - Use evaluative and iterative strategies
Target action	- Identifying and recruiting mother-daughter dyads to the 4GW Mother-Daughter - Day HPV campaign to increase uptake of HPV vaccination for girls and HPV screening for women
Temporality	- Within 6 months of trial enrollment, HPV vaccination and HPV screening uptake will be assessed
Dose	- Time of intervention: 3 months, 6 months
Implementation outcomes	- Implementation acceptability, appropriateness, feasibility, cost, fidelity, penetration and sustainment

#### Recruitment

We will enroll mother-daughter dyads in the 18 LGAS. Recruitment methods include in-person events and venue-based, participant referral, community, religious setting and school-based referrals, social media, and online referrals. First, mother-daughter dyads will be identified and recruited to participate in an upcoming Mother-Daughter Day event on HPV prevention methods. Second, those who agree to participate will receive reminder text messages before the event. Third, trained CHWs and supervisors will provide educational information and materials on cervical cancer control at the event.

Fourth, onsite services for HPV vaccination and self-collection kits will be offered to mother-daughter dyads, with girls receiving HPV vaccinations immediately in an onsite vaccination dedicated private space designated for the mother-daughter Day event. Mothers will receive instructions on self-collection in a private area, and their samples will be returned to the NIMR laboratory. Trained CHWs will provide the results of the samples, and those with positive results will be linked immediately to the supervisors for treatment and follow-up care, which we will track overtime. A baseline survey will be conducted among 612 mother/daughter dyads (*n* = 1,224 total) recruited in 18 LGAs (capped at 34 per area). We will define mothers as any female caregiver, including biological and surrogate mothers or close relatives who meet eligibility criteria. Eligibility criteria for mother-daughter dyads are outlined in Table 1. Trained community healthcare workers will approach and obtain consent from participants in a private space. Parental mobile phone numbers will be collected as tracking information for follow-up and retention.

#### Randomization and rationale for stepped-wedge design

**Fig. 2:**
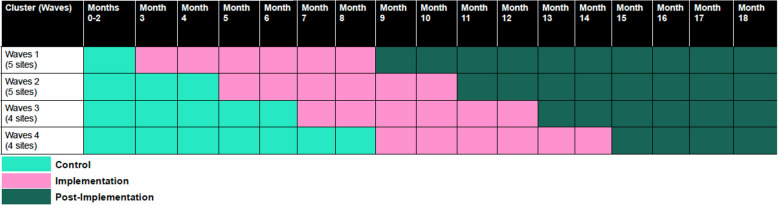
Stepped wedge study design

The 18 LGAs will be randomly allocated to the intervention, 4–5 LGAs per month for 12 months. All sites begin as part of the control condition and are block-randomized into four waves, with each wave beginning two months after the start of the prior wave and lasting for 6 months, followed by a 6-month follow-up period. All clusters will eventually receive the intervention, with outcomes measured every three months across all clusters. See Fig. 2for a schematic of the stepped-wedge cluster RCT design. Randomization will be conducted by the study statistician, who will not have direct contact with the LGAs. All LGAs will be informed of their assigned intervention wave before the first wave begins. Blinding was not implemented in this study, as all sites will eventually receive intervention, reflecting typical conditions in real-world settings. The stepped-wedge design was deemed acceptable and ethical by key program partners (NIMR and the Nigerian Ministry of Health) and study collaborators for logistical and pragmatic reasons, given the potential benefits of the intervention package (i.e., combined cervical cancer prevention strategies of vaccination and screening) and the equitable provision of the intervention to all sites [40].

#### Outcomes

Among daughters, the primary outcome will be HPV vaccination receipt (one dose) within 6 months of trial enrollment. Vaccines will be provided onsite at the Mother-Daughter Day campaign by study nurses following similar procedures for HPV vaccination during the national rollout [61]. Among mothers, the primary outcome will be self-collection completion within 6 months of trial enrollment, which will also be operationally defined by the receipt of specimens at the NIMR laboratory. As a secondary outcome, we explore HPV vaccine confidence, hesitancy and screening confidence, and the linkage to follow-up care and treatment of women with positive HPV results will be tracked. Using the validated Weiner's surveys, we will assess the acceptability and appropriateness of intervention components from both the daughters'and mothers'perspectives [62]. These surveys include 4 items, each scored on a 5-point scale [62]. Penetration will be explored using the Levels of Institutionalization Scale [63] to assess the extent to which components of the mother-daughter HPV campaigns are institutionalized within participating local government areas. We will also assess plans for sustaining [59] intervention components that will include 3-item leadership support for implementing the HPV campaign, a 3-item scale on responsiveness to mother-daughter preferences and needs for HPV preventive campaigns, an 8-item scale on coalitions, and partnerships to sustain the project, the 9-item infrastructure and capacity to support sustainability and the 3-item monitoring and evaluation tool from the sustainment measure on a 5-point scale [64]. We will also assess the intervention fidelity [65] and track adaptations made using the FRAME-IS [66] tool for reporting adaptation across the 18 LGAs.

#### Sample size and power considerations

We performed an a priori power analysis to determine the sufficient sample size for the stepped-wedge cluster RCT design, where the unit of randomization is the geographic area (cluster). Thus, there are two important sample estimates: N, for the number of mother/daughter dyads and K, for the number of LGAs. Specifically, the N participants will be nested within the K clusters. This nesting will likely create dependency on the data. The degree of this dependency depends on the intraclass correlation coefficient (ICC). The required sample size is calculated for the primary outcomes of HPV vaccination uptake for daughters and HPV self-collection uptake for mothers. Assuming a total of 18 LGAs with 34 dyads per LGA (N1 = 18 × 34 = 612 for daughters and N2 = 18 × 34 = 612 for mothers, total N = N1 + N2 = 1224), four total intervention periods, 2-sided alpha = 0.05 and 80% power, we calculated the minimum detectable rate of the primary outcome uptake at follow-up (Table 3 below). We expect three possible baseline rates of 0.10, 0.15, and 0.20 and compute the minimum detectable differences between the baseline and follow-up rates (hence minimum detectable follow-up rates) for the two primary study outcomes, assuming three possible ICC values of 0.005, 0.01, and 0.05. As shown in Table 1, the minimally detectable follow-up rates are much less than expected of the follow-up 50–60% self-collection uptake rates [67–69] and 65% HPV vaccination [11]. Hence, we have sufficient power (> 80%) to detect the increases in HPV vaccination among girls and HPV self-collection among their mothers after the implementation of the final crowdsourced campaign with a total of 18 clusters with 34 dyads per cluster (Table 3). To account for the 15% attrition rate at 6 months follow-up, we increased the sample size to 704 for mothers and daughters (40 dyads for each cluster). The calculation was performed using the software PASS 2019 (Power Analysis and Sample Size Software (2019) with the formulas developed by Hussey et al. [70]

**Table 3:** Power and sample size for stepped-wedge RCT^a^

Cluster size (total sample size)	Rate of primary outcome at baseline (HPV self-collection uptake for mothers, HPV vaccination for daughters)								
	0.10^a^			^a^0.15			^a^0.20		
	ICC			ICC			ICC		
	0.005	0.01	0.05	0.005	0.01	0.05	0.005	0.01	0.05
	Minimally detectable rate of primary outcome at follow-up			Minimally detectable rate of primary outcome at follow-up			Minimally detectable rate of primary outcome at follow-up		
18(612)	^b^0.216	^b^0.220	^b^0.235	^b^0.283	^b^0.285	^b^0.300	^b^0.342	^b^0.346	^b^0.362

^a^Rate of primary outcome at baseline

^b^Minimum detectable rate of the primary outcome at follow-up composed of HPV prevention services uptake among intervention arm (2-sided test, 5% significance level, statistical power of 0.80)

### Statistical analysis

#### Data analysis

The primary analyses will examine rates of HPV self-collection (mothers) and single-dose vaccine receipt (daughters). Analyses will be performed separately for the mother sample and daughter samples. The main analyses will compare the rates of HPV self-collection (mothers) and vaccine uptake during the pre-and post-intervention periods. We will examine a hypothesis comparing the superiority of the intervention with pre-intervention periods using the stepped-wedge cluster randomized control design and account for any temporal changes in HPV vaccination or screening rates during the 6-month conduct of the trial. This analysis will be accomplished with generalized linear mixed models to account for the within-person factor – time (baseline, 3 months, and 6 months) and one primary between-person factor (randomization site dummy code as 0 = convention and 1 = intervention). The 3-level model is structured as observations nested within subjects within local government areas. Multilevel modeling software (SAS, version 9.4, PROC GLIMMIX with logit as link function) will be used to compute full information maximum likelihood (FIML) estimates of the model parameters. The model will include intervention status and time as fixed effects and site and individuals as random effects. The estimated intervention effects will be reported with 95% CIs and p values. Secondary analyses will explore linkage rates to clinical follow-up after positive HPV testing in pre-intervention and post-intervention periods.

#### Implementation outcomes and process evaluation

Table 4lists the measures for our implementation outcomes and process evaluation. We will use a mixed-methods evaluation to explore why the final HPV campaign worked or did not work to increase the uptake of HPV vaccination among daughters and HPV screening among mothers. Proctor’s Implementation Outcomes framework guides this theory-based process evaluation to assess the intervention's perceived impact and underlying mechanisms of action [41]. We will collect three data sets: theory-informed measures, community healthcare worker ratings and observations to assess implementation fidelity, and in-depth interviews with participants, CHWs, and supervisors. The theory-informed measures, which include demographic questions plus validated surveys (with adaptations when necessary), are presented in Table 4with some addressing relevant constructs (e.g., implementation fidelity, penetration) related to intervention implementation. The measures will also explore outcomes that uncover the underlying mechanism through which the intervention may or may not have influenced intervention uptake as intended. The semi-structured interview guide will include questions assessing implementation fidelity by community healthcare workers according to the fidelity framework developed by Carroll and colleagues [65]. Interviews will last 45–60 min and will be recorded for transcription, coding, and analysis.

**Table 4:** Measures and outcomes for data collection

Construct	Measurement	Description	Timing of administration
Intervention Acceptability	Acceptability of Intervention Measure (AIM) Cronbach alpha = 0.85	Fifteen-item measures (3 subscales) of implementation outcomes that are often considered “leading indicators” of implementation success [71, 72] The subscales are rated on a 5-point Likert scale, 1 to 5, with higher scores indicating higher acceptability, appropriateness, and feasibility	Baseline, 3 months, 6 months
Intervention Appropriateness	Intervention Appropriateness Measure (IAM) Cronbach alpha = 0.91		
Intervention Feasibility	Feasibility of Intervention Measure (FIM) Cronbach aflpha = 0.89		
Adoption (mothers)	Uptake of HPV self-collection among mothers	The proportion of eligible women with completion of HPV self-collection	Baseline, 3 months, 6 months
Adoption (daughters)	Uptake of HPV Vaccination among Daughters	The proportion of eligible girls with HPV vaccination receipt (one dose)	Baseline, 3 months, 6 months
Penetration (i.e. Levels of Institutionalization Scale)	Penetration Cronbach alpha = n/a	The extent to which components of the mother-daughter HPV campaigns are institutionalized within participating LGAs	Baseline, 3 months, 6 months
Implementation fidelity	Evidence that all core intervention components were delivered as intended and an assessment of intervention implementation concerning the study protocol consistency of implementation across the 18 local government areas	More specifically, fidelity will be assessed both quantitively and qualitatively using the following four dimensions: (1) Frequency, number of intervention-related interactions; (2) Duration: length of each component of the intervention; (3) Content: the knowledge or behavioural change the combined intervention seeks to deliver to the mother/daughter dyads; and (4) Coverage: the number of mother/daughter dyads who receive the intervention as intended over the number of participants who are enrolled	Baseline, 3 months, 6 months
Identification of barriers to HPV vaccination and/or screening	Open-ended semi-structured questionnaire	Qualitative research will examine barriers to vaccination/screening and reasons for failure to complete vaccination/screening	Baseline, 3 months, 6 months
General	Socio-demographic Characteristics	Six items included participants'age, sex, education level, place of residence, parent’s education level, family composition, and structure	Baseline, 3 months, 6 months and 12 months
Implementation leadership support	Measured using the Sustainment Measurement System Scale [73]. Items will be rated on a 5-point Likert scale ranging from 1 (not at all) to 5 (all the time), with lower scores indicating lower levels of agreement while higher scores indicating higher levels of agreement	Three-item subscale measuring the active engagement of leaders and/or program champions in project implementation and sustainment	Baseline, 3 months, 6 months and 12 months
Responsiveness to mother/daughter preferences and needs for HPV preventive campaigns		The three-item subscale measures the needs of the target population being served and their consistency and fit with norms and values	
Existing coalitions and partnerships to sustain the project		Eight-item subscale measuring networking with community-based organizations and institutions committed to program sustainability	
Infrastructure and capacity to support sustainability		Seven-item subscale measuring available resources for project sustainment and integration into operations of the organization	
Monitoring and evaluation tool		Three-item subscale measuring ongoing evaluation of progress made toward sustainment	

#### Mixed methods implementation evaluation analysis

For quantitative surveys, internal consistency will be assessed for theoretical constructs. If internal consistency is < 0.7, we will explore whether we will omit individual items. We will then calculate the means of the items measuring each construct to create a summary score ranging from one to five. We will also calculate the means and standard deviation of all measured constructs and compute separately for each measured item. Qualitative data analysis (interviews, observations) will occur iteratively using thematic analysis informed by the approach of Braun and Clark [74] with six stages for thematic analysis: 1) familiarization of interview data; 2) production of initial deductive and inductive codes by multiple coders; 3) review generated codes for consensus among coders; 4) search for themes and explore relationship between codes; 5) revise and summarize themes using thematic mapping to explore relationship between themes and discuss with multiple teams; and 6) written report.

#### Data synthesis and triangulation

We will follow NIH best practices for a sequential mixed methods approach for data explanation. We will use the embedded qualitative data to elaborate on or contextualize quantitative results [71, 75]. Comparisons between constructs will allow us to use the qualitative data to explain quantitative findings. We will triangulate data that explores key questions regarding implementing the combined crowdsourced campaign. A synthesis of findings from the questionnaires and qualitative data will be used to highlight key mechanisms, the implementation process, and the study outcomes.

### Cost-effectiveness and budget analysis of the HPV campaign

We will conduct an economic evaluation of the finalist HPV campaign. Using a micro-costing approach, we will collect direct medical costs alongside the trial from a healthcare provider’s perspective, including the cost of providing HPV vaccination, cervical cancer screening, and costs related to the intervention development.

### Ethical considerations

The protocol was registered with Clinical Trials.gov, under registration NCT06728085.

## Discussion

The development of rigorous crowdsourced implementation science strategies is a critical need for the field of implementation science globally. This pragmatic hybrid effectiveness-implementation type II stepped-wedge cluster randomized control trial is a collaborative effort between researchers from the Washington University School of Medicine, University of North Carolina Chapel Hill, Monash University (Australia), Wake Forest University School of Medicine and the Nigerian Institute of Medical Research (NIMR) that responds to this need and is intended to strengthen the use of crowdsourced strategies to improve uptake of HPV vaccination and HPV screening in Africa. While explanatory trials seek to understand the benefit of an intervention under controlled conditions using carefully selected participants [72], pragmatic trials have fewer eligibility requirements and broader potential generalizability. We will test the hypothesis that a combined mother-daughter campaign implemented by CHWs and tailored and adapted to local contexts, with education on cervical cancer and onsite services provided (i.e. access to HPV vaccination and HPV screening in private spaces), increases HPV vaccination among girls and HPV self-collection among mothers in Nigeria recruited from 18 local government areas. The primary outcomes will be HPV preventive measures: HPV vaccination uptake (one dose) among girls, verified through clinic records, and the return of HPV self-collection kits among mothers, confirmed by laboratory receipt of self-collected specimens. The secondary outcomes are HPV vaccine confidence, vaccine hesitancy, linkage and receipt of follow-up care among women with positive (abnormal) HPV self-collection results and implementation outcomes guided by Proctors Implementation Outcomes Framework [41]. HPV vaccinations and HPV screening are evidence-based interventions to reduce the burden of cervical cancer. While the nationwide rollout of HPV vaccinations for girls has begun in Nigeria [61], there remains limited data on the nationwide rollout of HPV screening for women. Furthermore, in Nigerian culture, mothers primarily influence their daughter’s health behaviors, creating and supporting opportunities that influence their health. Despite the key roles that they play, most HPV prevention interventions in Nigeria have minimally involved both mothers and daughters in intervention design and implementation, despite the evidence to support its feasibility and acceptability.

This study will use a stepped-wedge cluster randomized trial to assess the impact of a crowdsourced, community-engaged mother-daughter campaign and implementation strategy bundle on HPV vaccination among girls and HPV screening among their mothers in Nigeria over 6 months in 18 Nigerian LGAs. This study will also inform how working with end-users enhances the uptake of HPV prevention interventions. In general, little prior research has focused on engaging both mothers and daughters in intervention design despite their being at high risk for cervical cancer. Our study, which focuses on the implementation strategies of a crowdsourced Mother-Daughter Day Campaign for HPV alongside implementation outcomes, may accelerate public health impact by facilitating replication, research-to-practice implementation, and design for implementation and sustainability. Similar studies conducted in Peru [11] and South Africa [16], found that implementing HPV vaccination and screening is feasible and can improve the simultaneous achievement of the research-to-practice translation of global targets of 90–70–90 (90% HPV vaccination coverage, 70% screening coverage, and 90% access to treatment for pre-cancerous and cancerous lesions among women) by 2030 [2]. Both interventions were designed with minimal input from mothers and daughters; however researchers noted that both HPV vaccination and HPV screening for mother-daughter dyads hold promise for improving cervical cancer prevention and that a simple health education during vaccine implementation improved screening behaviors when screening was easy to access [11, 16]. To overcome suboptimal implementation challenges, tailored participatory strategies that address critical barriers with end users are needed. Intervention design and pilot-testing of the final mother-daughter day campaign have also included mothers and daughters with lived experience on factors that influence the uptake of HPV vaccination for girls and HPV screening for women. Transparent reporting of implementation strategies and alignment with implementation outcomes will provide a clear picture for future scale-up of Mother-Daughter Day campaigns for cervical cancer control, eliminating missed opportunities and providing valuable information for replication in other contexts. Our 4GW trial will be among the first to generate evidence on the effectiveness of a combined HPV campaign for mothers and daughters, co-designed and implemented with their input.

In summary, innovative approaches to cervical cancer screening and vaccination services among mother/daughter dyads in LMICs are needed. Yet, many HPV interventions repackage old ideas that are not designed or delivered with the end-users in mind. Our study will inform future HPV preventive services in Nigeria, drawing on the creativity and power of Nigerian individuals and communities, including mothers and daughters, who are traditionally underrepresented in dissemination and implementation science research in LMICs. Our multi-disciplinary team and history of collaboration increase the likelihood of success and clear relevance for scale-up and replication of combined HPV vaccination and HPV primary screening programs in Nigeria and other LMICs.

## Supplementary Information

Additional file 1. Crowdsourced implementation strategy bundle.

Additional file 2

## Data Availability

The datasets generated and analyzed during this study will be made available upon reasonable request from the corresponding author. Access will be granted in line with institutional policies and ethical considerations, ensuring data privacy and security.
