# Supplementary figures and images for: For girls and women (4GW) HPV RCT protocol: a crowdsourced, pragmatic stepped-wedge cluster randomized trial to improve uptake of HPV vaccination and screening among mother-daughter dyads in Nigeria
Source: Implement Sci. 2025 May 1;20:18. doi: 10.1186/s13012-025-01428-5 (PMC12046803; doi:10.1186/s13012-025-01428-5)

**Additional File 1: Crowdsourced implementation strategy bundle**


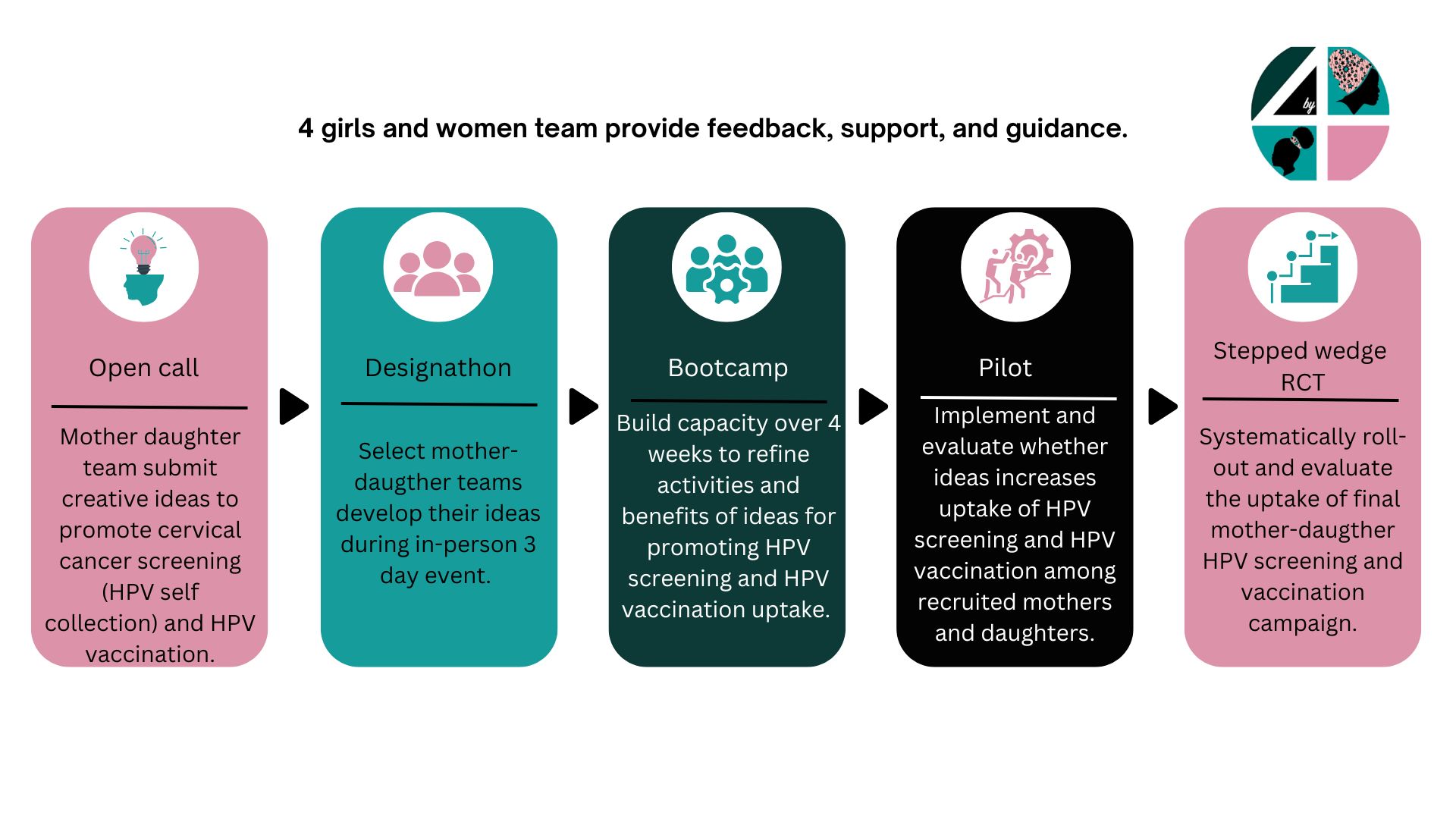

Supplement: Supplementary file 1 — Additional file 1. Crowdsourced implementation strategy bundle. [file 13012_2025_1428_MOESM1_ESM.docx]
